# A case report of zoster-induced Guillain–Barré syndrome: diagnostic challenges and potential role of pulse prednisone

**DOI:** 10.1097/MS9.0000000000003583

**Published:** 2025-07-16

**Authors:** Devrakshita Mishra, Omar Nayeem, Sameer Kumar Majety, Niyaz Shaik, Gopichand Muppana

**Affiliations:** a School of Medicine, Xiamen University, Xiamen, P.R. China; b Department of Internal Medicine, National Pirogov Memorial Medical University, Vinnytsia, Ukraine

**Keywords:** acute inflammatory demyelinating polyneuropathy, acute motor axonal neuropathy, corticosteroid therapy, herpes zoster, zoster-induced Guillain–Barré syndrome

## Abstract

**Introduction and importance:**

Zoster-induced Guillain–Barré syndrome (ZGBS) is a rare neurological complication of varicella-zoster virus (VZV) reactivation. Diagnosing ZGBS is challenging due to its overlapping clinical features with other forms of Guillain–Barré syndrome (GBS) and zoster myelitis. This report emphasizes the importance of early recognition and tailored treatment, particularly in resource-limited settings.

**Case presentation:**

A 45-year-old Indian male presented with a 10-day history of progressive lower limb weakness and paraesthesias. Physical examination revealed a maculovesicular rash in the left C8–T2 dermatomes, areflexia, and Grade 3 muscle strength in the lower limbs and distal upper limbs, indicating lower motor neuron involvement. Cerebrospinal fluid (CSF) analysis showed albuminocytologic dissociation, and nerve conduction studies confirmed motor axonal neuropathy, consistent with the AMAN subtype of GBS.

**Clinical discussion:**

The patient was initially treated with intravenous acyclovir for suspected herpes zoster myelitis but showed no improvement. Due to limited access to intravenous immunoglobulin (IVIG), pulse prednisone therapy was initiated. The patient required supplemental oxygen for mild respiratory distress during treatment. Prolonged prednisone therapy (11 days) resulted in significant clinical improvement, with full limb function restored within 7 days of therapy tapering and complete recovery achieved by day 18 post-treatment initiation.

**Conclusion:**

This case underscores the diagnostic complexity of ZGBS and highlights prolonged pulse prednisone therapy as a viable alternative to IVIG in resource-constrained settings. Early diagnosis and tailored management are critical for optimizing recovery in rare conditions like ZGBS.

## Introduction

Guillain–Barré syndrome (GBS) is an autoimmune condition that is often triggered after an infectious process, commonly a gastrointestinal or an upper respiratory infection, and rarely after viral infections of the human herpes family^[1]^. Among the former, GBS occurring after an eruption of zoster is rather a rare occurrence with an incidence of only 0.025% per 2 years^[2]^. The Clinical complex of GBS might vary with each patient but typically starts with sensorimotor dysfunction affecting lower limbs the earliest, which justifies the name, “Ascending Paralysis,” that is widely used in conjunction with GBS. Clinical manifestations include, but are not limited, to the following: progressive muscle weakness evolving over a period of a few hours to days that is associated with sensory dysesthesias in the extremities, facial diparesis (in about 50% of patients) which may lead to pooled oral secretion and associated complications, autonomic dysfunction which may present as postural hypotension or cardiac dysrhythmias, respiratory disturbances and pain in the neck, shoulder, back, or diffusely over the spine^[1]^.

**HIGHLIGHTS**

- Diagnostic challenges in zoster-induced GBS with overlapping zoster myelitis features.
- Rare mixed AIDP/AMAN subtype linked to herpes zoster reactivation and motor deficits.
- Immune dysregulation and molecular mimicry as potential mechanisms of ZGBS.
- Full recovery achieved rapidly within 18 days with extended corticosteriod therapy.
- Prolonged corticosteroid therapy as a viable option in resource-limited settings.

GBS has several recognized subtypes, classified based on the electrodiagnostic tests, including Acute Inflammatory Demyelinating Polyneuropathy (AIDP), the most common type, Acute Motor Axonal Neuropathy (AMAN), the most severe subtype and Acute Motor Sensory Axonal Neuropathy (AMSAN) subtype. Miller Fisher syndrome (MFS) is also a notable among the transient subtypes^[1]^. The diagnosis of GBS is typically made using the Brighton Criteria, which combines clinical symptoms, cerebrospinal fluid (CSF) analysis, and electrodiagnostic studies to classify cases into different levels of diagnostic certainty^[3]^. A characteristic finding in the cerebrospinal fluid (CSF) analysis for GBS is “albuminocytologic dissociation,” which refers to an elevated protein concentration in the CSF without an accompanying increase in cell count^[3]^.

The present treatment standard suggests the use of Intravenous Immunoglobulin infusions (IVIg) and Plasmapheresis (PLEX) as soon as the diagnosis is made, as studies prove early initiation of treatment is associated with a favorable clinical course^[1]^. IVIG infusions are administered as a 5 day daily infusion to meet a total dosage of 2 g/kg body weight, whereas PLEX is usually aimed to perform ~40–50 mL/kg plasma exchange (PE) 4–5 times over a period of 7–10 days^[1]^. The prognosis for patients with GBS is generally favorable, with approximately 85% achieving complete functional recovery within several months. However, some may experience persistent areflexia. In optimal healthcare settings, the mortality rate is below 5%, typically resulting from secondary pulmonary complications^[1]^.

This case report provides valuable insights into the diagnostic intricacies of zoster-induced GBS (ZGBS) and how clinicians can diagnose it in favor of other immune-mediated neuropathies as well as myelitis. Additionally, the report indicates that there could be a place for prolonged corticosteroid treatment as a resource-friendly alternative to IVIG. It also points to need for further treatment protocols based on individualization of post-viral immune neuropathies.

## Case presentation

The patient is a 45-year-old male who was admitted to the hospital on 9 October 2022. His primary complaint was weakness in both legs, which began 10 days ago and had been gradually worsening. Additionally, he had been experiencing tingling and numbness in his arms and legs, which had spread to his entire body over the past 10 days. He also had a history of chronic alcohol use and smoking.

**Figure 1.**
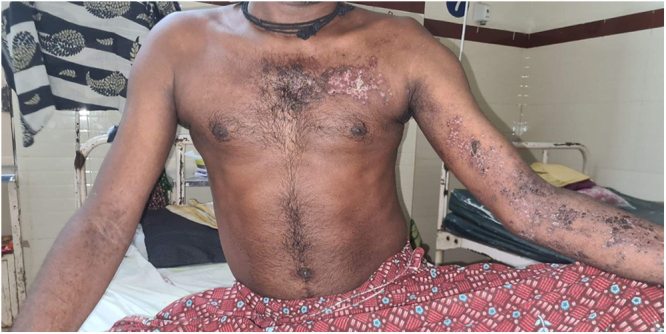
(A) Image of the patient showing maculovesicular rash along the C8 and T2 dermatomes on day 7 of admission. (B) Timeline of key events in patient management.

The patient did not report fever, chills, shortness of breath, chest pain, vomiting, diarrhea, neck pain, or back pain. The patient also has no history of trauma or similar complaints in the past. On examination, he exhibited a Grade 3 out of 5 muscle strength in both the proximal and distal lower limbs, as well as the bilateral distal upper limbs. The proximal upper limbs showed a muscle strength of Grade 4 out of 5. Additionally, a maculo-vescicular rash was observed, which the patient reported noticing about 5 days before admission. He described the rash as painful and itchy and mentioned that he had not sought medical help for it previously. Figure 1 shows the rash on Day 7 of admission, located along the left arm, forearm, and upper left chest, consistent with an active herpes infection in the C8, T1, and T2 dermatomes. Thus, diagnosis of the zoster infection was clinical in nature, due to the lack of sophisticated testing facilities at the hospital. When tested for Deep tendon reflexes, the patient demonstrated areflexia consistent with a Lower Motor Neuron (LMN) Disease.

The patient also reported a fall down the stairs attributed to lower limb weakness, necessitating the use of a walking cane. A second fall occurred due to a weak hand grip, rendering the patient unable to independently maintain the cane. Following this, the patient became bedridden, requiring assistance to get out of bed.

A CT scan of the spine was conducted subsequently, revealing a disc osteophyte complex at the C6–C7 level, causing indentation of the thecal sac. However, this finding was not consistent with cervical myelopathy, as other characteristic features of this condition were absent, it was ruled out as a diagnosis.

The next differential considered was zoster myelitis. As the paresthesia had already been present for over 10 days on admission, the patient was started on Acyclovir (Inj Acyclovir 800 mg IV TID) and Prednisone (1 gm in 500 ml of NS IV OD) for a period of 5 days. On observing no improvement after one complete course of treatment, the patient was sent to further undertake a Nerve Conduction Study (NCS) and a CSF analysis, the results of which have been shown in Table 1. The NCS showed a sensory and motor axonal neuropathy of the upper and lower limbs. Both consistent with a diagnosis of the AMAN subtype of GBS. The diagnosis was further confirmed with two Electrodiagnosic Criteriae, namely the Hadden Criteria and the Rajbally Criteria^[4]^. The CSF findings included mildly elevated protein levels with relative normal cell count and glucose levels.

**Table 1:** Nerve conduction study parameters and CSF findings of the patient

Nerve	Muscle	Latency (ms)	Amplitude (mV)	Conduction velocity (m/s)	F-wave latency (ms)	Interpretation
Right median (motor)	APB	Borderline normal (3.92)	Distal: 3.3, proximal: 3.0	Slowed (44.8)	Prolonged (30.2)	Features of AMAN with mild secondary demyelination (F-wave delay, mild slowing)
Left median (motor)	APB	Borderline normal (3.96)	Distal: 1.5, proximal: 1.4	Slowed (43.8)	Prolonged (32.8)	Axonal pattern
Right ulnar (motor)	ADM	Mildly prolonged (3.31)	Distal: 4.3, proximal: 3.9	Slowed (44.0)	Prolonged (32.2)	Axonal motor neuropathy with mild secondary slowing
Left ulnar (motor)	ADM	Prolonged (3.85)	Distal: 3.1, proximal: 1.1	Slowed (46.0)	Prolonged (NR)	Moderate axonal involvement; slowed velocity suggests secondary demyelination
Right peroneal (motor)	EDB	NR	NR	NR	NR	Severe axonal involvement, absent responses
Right tibial (motor)	AH	Normal (3.85)	Distal: 3.5, proximal: 2.0	Slowed (33.6)	Prolonged (57.8)	Severe axonal loss with secondary conduction slowing
Right median (sensory)	Index	Prolonged (3.39)	Reduced (4.1 µV)	Slowed (38)	—	Sensory nerves relatively preserved; mild slowing could reflect subclinical involvement
Right ulnar (sensory)	Dig V	Prolonged (2.45)	Reduced (3.4 µV)	Slowed (38)	—	Mild sensory slowing; consistent with secondary changes
Right sural (sensory)	Calf-ankle	Prolonged (2.92)	Low-normal (4.0 µV)	Borderline (43)	—	Preserved sensory conduction supports AMAN subtype with limited secondary involvement
**Cerebrospinal fluid (CSF) findings**						
**Parameter**					**Findings**	
Protein					52 mg/dL	
White blood cell (WBC) count					3 cells/μL	
Glucose					68 mg/dL	
Opening pressure					9 cm H_2_O	
Red blood cells (RBC)					Absent	
**Electro diagnostic parameter**			**Hadden *et al***^[4]^	**Rajbally *et al***^[4]^	**Our Case**	**Interpretation**
AIDP criteria met			DL >110% ULN, CB, TD, F-wave >120% ULN	Similar with stricter CB (P:D <0.7)	DL normal, no CB/TD, isolated prolonged F-wave	Does not meet AIDP criteria
CMAP amplitude			D-amp <80% LLN → AMAN	Same	Absent in peroneal, reduced in tibial	Consistent with AMAN
SNAPs			May be reduced in AIDP	Should be preserved in AMAN	All normal	Supports AMAN
Motor conduction velocity			<90% LLN in AIDP	<70% LLN in AIDP	Tibial ~32–33.6 m/s (borderline)	Suggests possible secondary slowing
Final diagnosis according to the criteria			AMAN	AMAN	Fulfills AMAN with mild delayed slowing	Primary AMAN with secondary demyelination

**Figure 2.**
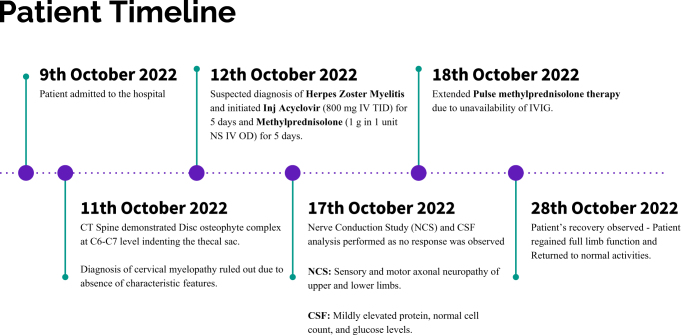
Timeline of Key Events in Patient Management.

Due to limited IVIg availability at the hospital, the pulse prednisone therapy was extended. During this period, the patient developed mild respiratory distress. The respiratory distress was not severe enough to warrant mechanical ventilation, and therefore, the patient was placed on supplemental oxygen. The patient had a clinical response with the extended pulse prednisone therapy, which was given for a total period of 11 days, including the first course, and then tapered off. He had a complete gain of limb function and returned to normalcy within the next 7 days (18th day post-initiation of therapy). Visual representation of the timeline of the patient is given in Fig. 2.

The patient was contacted for a follow-up after two and a half years from the initial admission. During the follow-up, a thorough history was taken, and a comprehensive examination was conducted. No notable findings were reported post-discharge. The patient’s immunization status was confirmed, revealing that he was unvaccinated for VZV/HZ. Additionally, the patient reported a history of chickenpox during childhood, although he could not recall the exact time period of the infection.

This case has been reported in line with scare criteria 2025^[5]^.

## Discussion

GBS is a post-infectious, immune-mediated neuropathy typically occurring following an infection, resulting in muscle weakness and, in severe instances, autonomic dysfunction. This condition arises from an immune response that aberrantly targets the peripheral nerves, and its subtypes exhibit distinct patterns on electrophysiological studies. The principal subtypes are acute inflammatory demyelinating polyradiculoneuropathy (AIDP), most prevalent in Western countries and characterized by demyelination with slowed nerve conduction; acute motor axonal neuropathy (AMAN), observed primarily in Asia and South America, which affects motor nerves and often has a more favorable prognosis due to the preservation of myelin; and acute motor sensory axonal neuropathy (AMSAN), a rarer and more severe variant involving both motor and sensory nerves, leading to significant axonal damage and slower recovery^[6]^. In this case, the diagnosis was consistent with AMAN, supported by nerve conduction studies that demonstrated both motor and mild sensory involvement, along with features of axonal loss and secondary demyelination.

GBS has been associated with a range of infections, with gastrointestinal and respiratory illnesses being the most frequently implicated. Notably, up to 70% of GBS patients report experiencing an antecedent illness within 1 to 6 weeks prior to symptom onset, underscoring a significant temporal association between such infections and the subsequent development of GBS^[7]^.

Numerous infectious agents have been implicated in the pathogenesis of GBS, with *Campylobacter jejuni* being the most prevalent, particularly with the AMAN variant of GBS . Other frequently identified etiological agents include *Epstein-Barr virus, Mycoplasma pneumoniae, Cytomegalovirus*, and HIV. Although rare, GBS has also been documented as a post-vaccination complication, particularly following vaccines for measles, rabies, influenza, and MMR. More recently, GBS cases have been reported in association with SARS-CoV-2 infection and COVID-19 vaccination, expanding the scope of known triggers^[8]^.

GBS is characterized by the immune system’s aberrant attack on the peripheral nervous system. Both innate and adaptive immune responses contribute to its pathogenesis. In AIDP, a primary subtype of GBS, research has demonstrated the presence of autoreactive CD4+ and CD8+ T cells targeting myelin proteins. These T cells, exhibiting a proinflammatory profile, are detected in the blood, cerebrospinal fluid, and nerve tissue. This observation strongly suggests their involvement in the demyelination process characteristic of AMAN. Conversely, in AIDP, a comparable T cell response has not been observed, indicating distinct immunopathological mechanisms underlying this form of the disease^[9]^.

Genetic predisposition also plays a role in GBS susceptibility and severity. Polymorphisms in genes such as tumor necrosis factor (TNF) and mannose-binding lectin (MBL) have been associated with increased risk and disease severity. Furthermore, the presence of autoantibodies directed against gangliosides, crucial components of nerve cell membranes, is a hallmark of GBS and contributes significantly to nerve damage^[9]^.

The progression of GBS can be rapid, with most patients reaching their peak level of disability within 2 weeks of symptom onset. Approximately 20% of GBS patients experience respiratory failure, necessitating mechanical ventilation. Additionally, involvement of the autonomic nervous system can lead to cardiac arrhythmias and blood pressure instability^[9]^. While recovery from GBS typically takes several months, this patient has made a remarkably swift recovery within just 20 days.

Acute varicella (chickenpox) infection is an infrequent trigger for GBS, with the condition typically manifesting as the AIDP subtype following varicella. A recent review found that 88% of GBS cases following varicella infection were of the AIDP type, while 13% were of an axonal variant. The pathophysiology of GBS associated with varicella infection remains inadequately understood, although several hypotheses have been suggested. These include molecular mimicry, in which the immune system mistakenly attacks peripheral nerves due to similarities between viral and nerve antigens, as well as immune dysregulation, particularly involving the host’s lymphocyte subtypes^[3]^. This case represents a very rare instance of GBS induced by varicella-zoster infection of the AMAN subtype, with no prior history of gastrointestinal or respiratory infections.

The process of molecular mimicry involves a T cell receptor recognizing both a viral and a self-antigen, thereby inducing an autoimmune response. Unlike nonspecific autoimmune mechanisms, molecular mimicry involves microbial mimics that specifically target the immune response to particular tissues or organs^[10]^. To establish molecular mimicry as the cause of an autoimmune disease, four key criteria must be met: structural similarity between host and microbial or environmental epitopes, cross-reactivity of antibodies or T-cells, an epidemiological association between exposure and disease onset, and reproducible induction of autoimmunity in animal models after being exposed to the relevant epitopes, through infection or environmental contact^[11]^.

GBS is a well-recognized example of an autoimmune disease triggered by molecular mimicry, where an immune response to viral antigens leads to cross-reactivity with host nerve tissues. In the case of ZGBS, reactivation of the varicella-zoster virus (VZV) may induce an aberrant immune response, generating autoantibodies that mistakenly target peripheral nerve components, leading to demyelination or axonal damage^[12]^.

However, not all individuals who experience viral infections develop post-infectious autoimmunity, suggesting that genetic predisposition, immune regulation, and pre-existing conditions influence susceptibility. Recent studies indicate the HLA subtypes linked with autoimmune conditions can modify immune responses after viral infections, making it more probable to have an abnormal immune response^[13]^. Older patients and immunocompromised patients can also have hyperactive immune states persisting for longer periods, increasing the risk of nerve injury due to autoantibodies further^[14]^. All these points highlight the need for individualized risk evaluation in patients after recovery from any viral infections; to determine those at higher risk of post-infectious neurological sequelae.

The occurrence of GBS following Shingles is a quite rare phenomenon occurring only in 0.025% of patients with Shingles based on a study with a sample size of more than 1 250 000^[15]^. This study also indicates that the adjusted hazards risk of developing GBS for a person with a herpes zoster infection is 17 times greater than a patient without infection^[15]^. It is to be noted that less than 5 similar cases to ours have been reported from India and neighboring countries in PubMed, indicating either rarity or under reporting of this instance. However it is also worth mentioning that an infection of VZV occurring primarily, i.e., Chicken Pox has resulted in higher number of patients being affected by GBS, that was evidenced by a study conducted by Islam *et al*^[16]^. This study also includes a systematic analysis of reported GBS cases following zoster infection, which found that all cases were demyelinating variants. In contrast, our patient demonstrated the AMAN variant, highlighting a rare axonal presentation in this context^[16]^. The pathogenesis of this instance is poorly understood due to lack of existing research on the exact pathogenesis of GBS post Shingles and how it might be different.

The diagnosis of GBS is usually done based on Brighton criteria, as mentioned previously, which utilizes both clinical and laboratory finding to ascertain the diagnosis^[3]^. The differentials considered in our case were wide due to lack of the usual precipitant causes in the history combined with presence of rash and imaging findings, including zoster myelitis and cervical myelopathy. Computed tomography findings in our cases showed indentation of the thecal sac at the C6–C7 level, however the patient lacked the characteristic findings of cervical myelopathy, this led to ruling out of this diagnosis. Due to the presence of vesicular rash and motor weakness, the diagnosis of zoster myelitis or segmental motor weakness due to involvement of the spinal cord was thought. Zoster myelitis is widely recognized yet a rare complication associated with reactivation of VZV. Zoster myelitis can have a broad range of presentation as it can involve various regions of spinal cord like anterior or posterior spinal roots, the anterior or posterior spinal horns^[17]^. These findings may be confirmed by MRI and/or electrophysiological investigations^[17]^. The above mentioned MRI findings typically include hyperintense T2-weighted and T2-weighted FLAIR images and may demonstrate intense enhancement post-contrast administration^[18]^.

Electrophysiological findings in zoster myelitis often resemble those of other myelitis cases, typically showing reduced sensory nerve action potentials (SNAPs), decreased compound muscle action potentials (CMAPs), and impaired motor unit action potential (MUAP) recruitment in weakened muscles^[19]^. Similarly, AMAN-GBS patients typically exhibit reduced compound muscle action potential (CMAP) amplitudes with preserved sensory nerve action potentials (SNAPs), reflecting selective involvement of motor axons. F-wave responses may be absent or prolonged due to proximal conduction failure, particularly at the anterior roots. However, conduction velocities and distal motor latencies usually remain within normal limits, and temporal dispersion is not observed^[20]^. These electrophysiological features may overlap with early myelitis, making clinical correlation and additional investigations such as MRI and CSF analysis essential. Several standardized electrodiagnostic criteria exist to classify GBS variants. The Hadden criteria (1998) and the modified Rajabally criteria (2014) are among the most widely used^[4]^. Based on both frameworks, our patient meets the diagnostic features of AMAN (Table 1) with secondary demyelination.

Anti-ganglioside antibodies are critical biomarkers in the diagnosis of GBS and its variants, Miller Fisher syndrome (MFS) and acute motor axonal neuropathy (AMAN). Anti-ganglioside antibodies bind to gangliosides present in peripheral nerves, leading to immune-mediated damage. Detection methods like ELISA and immunodot assays detect specific antibodies, making subtype identification possible and determining treatment. Their detection is certain clinical diagnosis, although standardization of testing is a challenge^[21]^. However, this testing was not performed for our patient due to its unavailability at the hospital.

The clinical symptoms of zoster myelitis include bilateral symmetric weakness in lower limb and also might be associated with sphincter involvement, mimicking the ascending paralysis and autonomic dysfunction of GBS^[6]^. Although GBS following a zoster eruption has a relatively serious course of disease, zoster myelitis has a favorable prognosis with a complete recovery in 55–75% of cases when given prompt treatment with 14 day course of acyclovir with a 5-7 day course of corticosteroids^[17]^.

It is of paramount importance for a clinician to carefully examine the patient, administer suitable diagnostic tests and initiate the appropriate treatment as early as possible to minimize the complications. This is especially tested when a disease is indolently mimicking the other as explained above, our patient was initially treated empirically with Acyclovir and corticosteroids in suspicion of zoster myelitis. This was ruled out when the patient underwent other diagnostic tests like lumbar puncture and nerve conduction studies due to absence of clinical response with the first course of drugs. Only with the NCS was the diagnosis of GBS confirmed, warranting immediate initiation of targeted treatment, considering the patient had already developed mild respiratory distress.

The current treatment modalities of GBS can be divided into two groups: general supportive treatments and targeted therapy. Supportive care is considered as one of the most important treatment modalities in GBS cases as up to 30% of them can progress to respiratory failure^[22]^. The patient may be transferred to an intensive care unit to allow prompt and regular monitoring of various parameters, including respiratory, autonomic and bulbar functions^[23]^. Respiratory support includes mechanical ventilation in the form of supplemental O2 therapy, intubation or a tracheostomy, indicated if pulmonary function hasn’t been restored after 2 weeks of intubation^[24]^. A nasogastric or orogastric tube is indicated in patients with severe dysphagia. For patients with sensory symptoms like burning pain, marked radicular back pain or any neuropathic pain, that is refractory to treatment with NSAIDs, treatment with other pain-modulating drugs like antidepressants, pregabalin, gabapentin, tramadol, carbamazepine, etc., is indicated^[24]^.

Considering that these patients, particularly those with GBS, may remain bed-ridden for a prolonged duration, appropriate measures for prophylaxis against deep vein thrombosis (DVT) and muscle contractures are essential. DVT can arise from a confluence of factors, collectively known as Virchow’s triad: venous stasis, endothelial injury, and hypercoagulability. Prolonged immobility, a consequence of bed rest, directly contributes to venous stasis, thereby increasing the risk of thrombus formation. DVT prophylaxis can be achieved through various methods, including the use of compression stockings to promote venous return and/or the administration of anticoagulants, such as subcutaneous heparin, to inhibit clot formation^[22]^. Muscle contractures can be prevented by passive targeted movement of muscles on the bedside. Clinicians should also stay alert toward development of any pulmonary or urinary infections as well, commonly seen in severe cases of GBS. Patients are often alert and cognitively capable during this period; clinicians should be vigilant of the same. Even though the patient developed mild respiratory distress on day 5, no mechanical ventilation was necessary. The patient’s oxygen needs were supported with supplemental oxygen.

The current AAN (American Academy of Neurology) approved treatments for GBS include Plasma Exchange (PE) monotherapy and IVIG monotherapy. They recommend PE for – nonambulant adult patients who seek treatment within 4 weeks of onset of neuropathic symptoms, ambulant patients examined within 2 weeks of onset. IVIG is recommended for nonambulant adult patients within 2 or 4 weeks of onset of neuropathic symptoms. PE and IVIG may also be used in children with severe GBS. They also recommend against use of sequential treatment with PE followed by IVIG, and the use of corticosteroids as a form of management^[25]^. Our patient falls under the category of a non-ambulant adult patient seeking treatment within 2 weeks of onset of neuropathic symptoms and therefore, should have been given IVIG. However, due to limited availability, the hospital was unable to provide IVIG for this patient.

Intravenous corticosteroids are presently not recommended as a treatment or management modality in GBS. Multiple studies have shown them to be of no use or worse than the patients under no medication (control)^[22]^. However, all of these studies and findings are with regards to short courses of prednisone therapy, IV Prednisone 500 mg/day for 5 days each time. Although current evidence on corticosteroid use in GBS remains inconclusive, Prednisone was administered at the doctor’s discretion, as no other approved treatments (IVIg or PLEX) were available. The decision was further influenced by the patient’s mild respiratory distress and lack of improvement following a course of Acyclovir and Prednisone, prompting the need for an alternative approach. Notably, studies have shown that high-dose glucocorticoids (GCs) in AIDP patients are associated with shorter hospital stays (*P* = 0.023), lower Hughes scores at nadir (*P* < 0.001), at discharge (*P* = 0.005), and three months post-onset (*P* < 0.001) compared to lower doses, suggesting a potential benefit in the short-term recovery of AIDP patients. However, this effect was not observed in AMAN patients, where outcomes remained similar across different GC doses^[26]^. Furthermore, moderate-quality evidence from two large trials involving 467 participants found a slight improvement in disability after 4 weeks with intravenous corticosteroids, though the results still allowed for the possibility of no effect^[27]^. Given these mixed findings, the use of corticosteroids in this case was guided by pragmatic considerations, prioritizing symptom management in the absence of standard therapies.

Pulse Prednisone therapy was used as a replacement for IVIg, Inj Prednisone gm in 1 unit of NS IV OD. While the initial treatment course was 5 days, once the patient developed respiratory distress, along with the supportive care, the patient was given this dosage for another 6 days, making a total period of 11 days. The patient showed positive clinical response on day 12, As mentioned previously. The usual course of the disease post-treatment is on average 3-6 months to regain independent ambulation and full gain of function. However, in our case, the patient gained full function within 20 days. In addition to being a case of ZGBS, the duration of the treatment administered is also a significant variable in this instance, and may be deduced as one of the causes for the unusually short course. As in other autoimmune conditions, one of the mechanisms through which this occurred might be the corticosteroid inducing transient leukopenia and altering the lymphocyte recirculation^[28]^. It may be hypothesized that perhaps a 5-day treatment period is insufficient in cases of GBS, perhaps a longer period is necessary, the underlying mechanisms need to be studied further in depth.

Another treatment option that has been under study for GBS is the usage of Neurotrophic agents such as, Brain-derived neurotrophic factor (BDNF). Neurotrophic factors are a family of proteins that promote the growth, survival, and differentiation of neurons by interacting with specific receptors and triggering intracellular signaling pathways. They are important for maintaining neuronal function, enhancing synaptic plasticity, and facilitating nerve regeneration following injury^[29]^. As GBS is associated with immune-mediated demyelination and axonal damage, neurotrophic factors such as nerve growth factor (NGF) and brain-derived neurotrophic factor (BDNF) may facilitate remyelination, survival of remaining neurons, and axonal regeneration. However, a systematic review studying clinical trials studying the efficacy of various treatments for GBS concluded that there is no significant difference between placebo arm and patients treated with BDNF^[30]^. The generalizability of this study is somewhat limited, as it is based on a small sample size of only 10 participants. This is indicative of the need for wider, more extensive studies in order to truly comprehend the role of neurotrophic factors.

The overall prognosis of GBS has improved significantly with advances in treatment, such as IVIG and plasmapheresis, particularly in the early stages of the disease. Despite the availability of these therapies, the outcome can vary depending on a number of factors including severity of the condition at onset, age of the patient being older than 57 years, autonomic dysfunction, need for ICU, and the need for mechanical ventilation^[31,32]^. Studies suggest that most individuals with GBS experience a partial to full recovery, with 60–80% regaining independent ambulation within 6 months^[32]^. However, about 10–20% of patients may have long-term residual effects, including chronic pain, weakness, or fatigue^[32]^. The prognosis of AMAN has improved significantly with early recognition and timely initiation of treatment such as intravenous immunoglobulin or plasmapheresis. Although AMAN was previously thought to carry a poorer prognosis compared to AIDP, recent evidence suggests that many patients can achieve substantial or even complete functional recovery when treated early. Recovery in AMAN may vary depending on the severity of axonal loss. Some patients recover within weeks, while others with more extensive involvement may take several months to years to regain baseline function. In our case, the patient’s clinical course was favorable following prompt diagnosis and treatment, which supports the growing evidence that early intervention is critical for improving outcomes in axonal variants of GBS^[33]^. In evaluating recovery outcomes in GBS, functional scoring systems provide an objective framework to assess patient progress over time. The GBS disability score is a standardized tool commonly used to evaluate functional status and recovery in GBS patients, ranging from 0 (healthy) to 6 (death), and is helpful in tracking improvement or guiding rehabilitation. Although widely used in clinical studies to monitor longitudinal recovery and treatment response, this scale was not employed in the present patient due to the retrospective nature of the documentation^[21]^.

ZGBS is a rare, but serious, complication of varicella-zoster virus (VZV) reactivation. While most patients with ZGBS experience some level of recovery, the prognosis is variable. Studies indicate that approximately a subset of patients fully recover, while others may experience residual symptoms, including muscle weakness or post-herpetic neuralgia (PHN)^[34]^. The recovery in ZGBS tends to be slower compared to other GBS subtypes, with some individuals taking months to regain full function^[35]^. Factors influencing the prognosis include the severity of the herpes zoster infection, the degree of demyelination in peripheral nerves, and the timeliness of antiviral and immunomodulatory treatments. Older patients, in particular, may face a more challenging recovery due to the risk of persistent pain or neurological deficits, emphasizing the need for early intervention and supportive care^[36]^.

In this case, the patient was diagnosed with the AMAN subtype of GBS following zoster reactivation. As mentioned above, ZGBS has a slower recovery period, in months to a year, while the recovery in AMAN is case dependent, from rapid to variable. Despite this the patient showed remarkably rapid improvement, with full functional recovery within 18 days. While the prognosis for ZGBS varies, the patient’s recovery highlights the importance of early intervention with immunomodulatory therapies, even in mixed subtypes, and suggests that prolonged corticosteroid therapy may be an effective treatment strategy in improving the prognosis for ZGBS.

Distinguishing between rapidly progressing GBS and the early stages of Chronic Inflammatory Demyelinating Polyradiculoneuropathy (CIDP) can be difficult. Both conditions present with similar symptoms characterized by distal and proximal muscle weakness accompanied by sensory disturbances, elevated protein levels in spinal fluid without cell count changes, and nerve conduction studies demonstrate evidence of demyelination. The key distinction is the disease’s progression; GBS develops quickly with a rapid onset followed by a self-limiting course, while CIDP progresses slowly and persists chronically with a progressive or relapsing pattern^[37]^. Clinical guidelines, based on randomized controlled trials, advise against oral corticosteroids for GBS and weakly recommend against intravenous methylprednisolone, due to lack of benefit and potential harm. However, oral corticosteroids remain the first-line maintenance treatment for CIDP patients^[38]^.

CIDP was excluded as a differential diagnosis because the patient showed significant recovery within three weeks of admission, regaining limb function. Nerve conduction study (NCS) findings further supported this distinction, as diffusely slowed conduction velocities with reduced CMAP amplitudes are more characteristic of AMAN than chronic inflammatory demyelinating polyneuropathy (CIDP). In CIDP, sensory involvement is typically more pronounced, whereas this patient showed only mild sensory abnormalities. Moreover, the absence of features such as conduction block and non-uniform slowing, which are hallmark indicators of CIDP, favored a diagnosis of AMAN with secondary demyelination^[39]^. The diagnosis of AMAN was confirmed further, as mentioned previously, by the Hadden & Rajbally criteria for GBS (acute onset areflexic weakness developing over 4 weeks, along with the Electrodiagnostic features mentioned above)^[4]^. The criterion of duration for CIDP (>8 weeks duration) and the lack of relapsing-remitting course or proximal-predominant weakness further, confirmed through a follow up, excluded it as a possible diagnosis. Moreover, Antiganglioside Antibody testing can also be used to distinguish between patients of CIDP and GBS as well. CIDP patients test negative, while GBS patients test positive, as discussed previously.

## Limitations

One notable limitation of this case report is the limited availability of intravenous immunoglobulin (IVIg) and plasmapheresis (PE) in resource-limited settings, which influenced treatment decisions and may have impacted patient outcomes. Furthermore, in this resource-limited setting, diagnostic tests such as antibodies against the varicella zoster virus, were unavailable, hindering the ability to confirm the etiology definitively. Another limitation is the lack of serial electrophysiological studies to monitor recovery, which could have provided further insights into the disease course. Additionally, the absence of serological confirmation of VZV reactivation and ganglioside antibody testing may limit definitive conclusions on the immunopathogenesis in this case.

Additionally, the restricted access to more advanced diagnostic tools, such as MRIs, and the inability to confirm findings with PCR testing, further constrained the diagnostic process. Since this is a single-patient retrospective analysis, the applicability of such findings is inherently limited. One significant limitation was that some crucial data – the original nerve conduction study (NCS) tracings – was lost due to long-term archival gaps. Irretrievable loss of initial records, a problem shared by several retrospective studies. These factors highlight the challenges of managing GBS in resource-constrained settings and underscore the importance of early intervention with the available diagnostic and therapeutic options. Despite these limitations, this case provides valuable insights into ZGBS and emphasizes the need for further research involving larger, multicenter cohorts and access to advanced diagnostics to make more definitive conclusions. Future studies should explore alternative therapeutic strategies and address the gaps in diagnostic availability to improve outcomes for patients in similar settings.

## Conclusion

Despite being an uncommon occurrence, the link between varicella infection and GBS remains poorly understood, creating diagnostic and therapeutic challenges. Clinicians should maintain a high index of suspicion for ZGBS, particularly in patients presenting with recent herpes zoster infection and concomitant neurological symptoms, even in the absence of a classic ascending paralysis. This is crucial as the clinical presentation of zoster myelitis can overlap with ZGBS, potentially leading to diagnostic delays. Prompt recognition and initiation of appropriate management are essential for optimal outcomes, as delayed diagnosis and treatment can significantly worsen the prognosis in GBS. Further research is warranted to investigate the efficacy and optimal dosing of pulse Prednisone therapy in the management of ZGBS. This therapeutic approach may offer potential benefits in mitigating the inflammatory response and improving clinical outcomes.

## Data Availability

The data regarding the patient can be submitted by the corresponding author upon reasonable request.

## Research registration unique identifying number (UIN)

Not available for the case report.
